# The Blood Supply of the Stomach: Anatomical and Surgical Considerations

**DOI:** 10.3390/diagnostics15222896

**Published:** 2025-11-15

**Authors:** George Triantafyllou, Orestis Lyros, Dimitrios Schizas, Nikolaos Arkadopoulos, Fotis Demetriou, George Tsakotos, Alexandros Samolis, Maria Piagkou

**Affiliations:** 1 Department of Anatomy, School of Medicine, Faculty of Health Sciences, National and Kapodistrian University of Athens, 11527 Athens, Greece; georgerose406@gmail.com (G.T.); fotisdemetriou2000@gmail.com (F.D.); gtsakotos@gmail.com (G.T.); alexsamolis@me.com (A.S.); 2 ”VARIANTIS” Research Laboratory, Department of Clinical Anatomy, Mazovian Academy in Plock, 09-400 Plock, Poland; 3 Fourth Department of Surgery, Attikon University Hospital, School of Medicine, Faculty of Health Sciences, National and Kapodistrian University of Athens, 12462 Athens, Greece; lyrosorestis@gmail.com (O.L.); narkado@hotmail.com (N.A.); 4 First Department of Surgery, Laikon University Hospital, School of Medicine, Faculty of Health Sciences, National and Kapodistrian University of Athens, 11527 Athens, Greece; schizasad@gmail.com

**Keywords:** stomach supply, gastric vessels, gastroepiploic vessels, variation, gastrectomy, bariatric surgery, surgical anatomy

## Abstract

The vascular anatomy of the stomach is both complex and highly variable, with direct implications for oncologic, bariatric, esophageal, and interventional procedures. This comprehensive review combines anatomical, radiological, and surgical evidence on arterial and venous variations in the stomach. The left gastric artery, traditionally the first branch of the coeliac trunk, often shows variants such as a direct aortic origin or association with an abnormal left hepatic artery. The right gastric artery most frequently arises from the proper hepatic artery, but its origin can vary significantly. The gastroepiploic arteries exhibit diversity in their origin, size, and connection patterns, with occasional duplication or absence. Additional vessels, including the posterior gastric artery and the short gastric arteries, also contribute to variations in arterial supply. Venous drainage largely follows the arterial pattern. The left and right gastric veins and the gastroepiploic venous arcade are major routes, while variants of the left gastric vein and the gastrocolic trunk (Henle’s trunk) contribute to complexity through different convergence patterns. These vascular variations have significant clinical implications, as they impact the safety of D2 lymphadenectomy, the risk of ischemic complications during laparoscopic sleeve gastrectomy, the success of gastric conduit formation in esophagectomy, and the effectiveness of transarterial embolization for upper gastrointestinal bleeding. Preoperative vascular mapping with multidetector computed tomography angiography and 3D reconstruction reliably defines individual anatomy, allowing for customized surgical planning and reducing operative risks. Recognizing both common and rare gastric vascular variants is essential for safe and effective surgical and endovascular management of gastric disease.

## 1. Introduction

The stomach is a part of the upper gastrointestinal system and lies between the esophagus and the duodenum. Topographically, it is situated in the left upper quadrant and extends anteriorly and to the right, occupying the left hypochondrium and epigastrium. The stomach is divided into the fundus, body, pyloric antrum, and pylorus. Moreover, adjacent to the esophageal opening, another region is the cardia [1]. Other important landmarks of the stomach include the lesser curvature, which extends between the cardiac and pyloric orifices and forms the medial border of the stomach. The greater curvature is longer than the lesser and extends from the cardiac notch to the pylorus. The gastrosplenic ligament and greater omentum attach laterally to the greater curvature [1]. The posterior surface of the stomach has important topographic relationships with the left crus of the diaphragm, the left inferior phrenic vessels, the left kidney, the anterior surface of the pancreas, and the upper layer of the transverse mesocolon [1].

The arterial supply of the stomach is predominantly derived from the coeliac trunk (CeT), one of the principal branches of the abdominal aorta (AA). Typically, the CeT gives rise to the left gastric artery (LGA), the common hepatic artery (CHA), and the splenic artery (SA). The LGA courses anteriorly into the lesser omentum, adjacent to the superior end of the lesser curvature, situated between the two peritoneal folds of the lesser omentum [1]. The CHA subsequently bifurcates into the proper hepatic artery (PHA) and the gastroduodenal artery (GDA). The second artery within the lesser omentum is the right gastric artery (RGA), which originates from the PHA and courses anteriorly into it, passing above the first part of the duodenum, while forming an anastomotic network with the LGA [1]. Furthermore, the right gastroepiploic artery (RGEA) branches from the GDA posterior to the first part of the duodenum and anterior to the pancreatic head. It courses laterally along the greater curvature and terminates in an anastomosis with the left gastroepiploic artery (LGEA), a branch of the SA [1].

The venous drainage of the stomach flows into the portal vein (PV) and is consistent and connected with the arteries. The gastric veins, specifically the left and right gastric veins (LGV and RGV), drain blood from the lesser curvature. In contrast, the left and right gastroepiploic veins (LGEV and RGEV) drain blood from the greater curvature [1]. These veins later drain into either the splenic vein (SV) or the superior mesenteric vein (SMV); however, they can also directly empty into the PV [1].

Beyond the macroscopic arterial and venous anatomy, the gastric mucosa exhibits a characteristic subepithelial capillary network. Scanning electron microscopy studies have shown a honeycomb-like capillary architecture mirroring the gastric pits [2]. High-resolution endoscopy further reveals distinct patterns: a honeycomb appearance in the body and a coil-shaped network in the antrum [3]. These networks are not only physiologically relevant for nutrient exchange and acid buffering, but also provide diagnostic markers, as disruption of the subepithelial capillary network is characteristic of early gastric cancer. Moreover, subepithelial tumors demonstrate distinct perfusion signatures on contrast-enhanced ultrasound, further emphasizing the clinical value of understanding gastric subepithelial vascularization [4].

The clinical and surgical anatomy of abdominal vessels is of utmost importance for anatomists, radiologists, and surgeons engaging with the abdominal cavity. Cadaveric and radiological research have examined the variations in these vessels in comprehensive detail, with direct implications for interventional procedures. Evidence-based meta-analyses have systematically reviewed these findings, emphasizing the increased morphological variability of these vessels [5,6,7,8,9,10,11,12,13,14].

Understanding the vascular anatomy of the stomach is crucial for anatomists, radiologists, and surgeons. Both cadaveric and imaging studies have documented significant variability, carrying major implications for surgical and interventional procedures. This review summarizes the current evidence regarding the blood supply of the stomach and its variations, with a focus on surgical relevance in procedures such as gastrectomy and bariatric surgery. A comprehensive literature search was conducted through PubMed, Web of Science, and Scopus up to August 2025, and the findings were critically evaluated to support clinical application. Anatomical, radiological and surgical studies recording the typical and variant vascular anatomy of the stomach were considered eligible.

## 2. Arterial System of the Stomach and Its Variations

### 2.1. Left Gastric Artery

**Figure 1:**
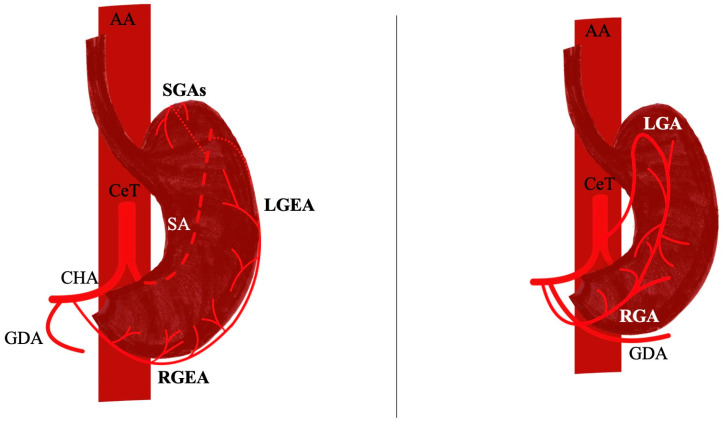
The typical arterial supply of the stomach with the left and right gastric and gastroepiploic arteries (LGA, RGA, LGEA, RGEA). CeT—coeliac trunk, CHA—common hepatic artery, SA—splenic artery, SGAs—short gastric arteries, GDA—gastroduodenal artery, AA—abdominal aorta.

The LGA is considered the first and smallest branch of the CeT, coursing superiorly toward the gastroesophageal junction before turning anteriorly along the lesser curvature of the stomach. Along its path, it provides esophageal and gastric branches and establishes anastomoses with the RGA. Its course makes it a critical landmark in gastric surgery, particularly since lymph node station 7 is located along the artery and is routinely dissected during D2 lymphadenectomy [1] (Figure 1).

**Figure 2:**
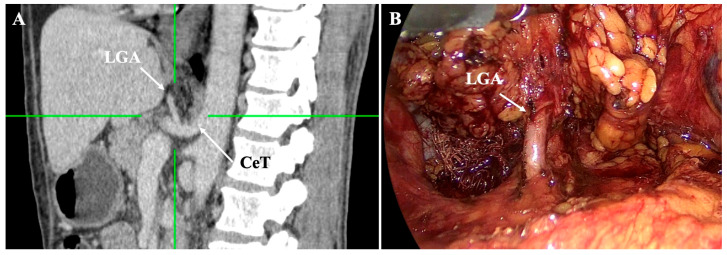
Visualization of typical left gastric artery (LGA) origin from the coeliac trunk (CeT) with computed tomography (**A**) and intraoperatively (**B**). Personal archive of Assistant Professor Orestis Lyros.

In the majority of cases, the LGA originates from the CeT as part of the classical trifurcation into the LGA, CHA, and SA (Figure 2). Meta-analyses of imaging and cadaveric studies confirm that this typical pattern occurs in approximately 75–83% of individuals [11,15]. However, the LGA demonstrates a wide range of anatomical variations in its origin and branching pattern. One of the most frequent variants is a direct origin of the LGA with the AA. This has been reported in both imaging and cadaveric studies, with pooled prevalence around 6–7% [11,15]. Less common origins include the participation in trunk variations such as a gastrosplenic trunk (with the CHA from the AA) [11,15]. Rarely, the LGA has been described as arising from the SMA or as absent altogether [11,15,16].

Duplication of the LGA is another documented variant. Mu et al. [17] reported cases in which two left gastric arteries arose from a short common trunk and diverged separately toward the stomach. Huang et al. [16] also described cases of dual LGAs branching directly from the CeT, as well as one case of LGA absence. Overall, studies report variation in the coeliac axis and its branches in approximately one-third of cases, with LGA anomalies comprising a minority but clinically relevant fraction [11,15]. These findings underscore that, although the LGA usually arises as the first branch of the CeT, its origin and number may differ significantly.

Another important anatomical variation is the presence of an aberrant left hepatic artery arising from the LGA. This anatomical possibility is relatively frequent with a pooled prevalence of 13.52% [6]. A replaced variant was estimated at 8.26% and an accessory one was recorded in 5.55% [6].

### 2.2. Right Gastric Artery

The RGA is a relatively small but consistent vessel that supplies the lesser curvature of the stomach. It usually runs within the lesser omentum, sending fine anterior and posterior branches before anastomosing with the LGA to complete the vascular arcade of the lesser curvature [1] (Figure 1).

The RGA is described as a branch of the CHA or, more commonly, the PHA. However, recent systematic investigations demonstrate that its origin is highly variable [18]. A meta-analysis of 15 anatomical and radiological studies including 1971 arteries found that the RGA most frequently arises from the PHA, with a pooled prevalence of 53.6%. The left hepatic artery accounted for the second most common origin (25.9%), followed by the GDA (8.9%). Less frequent origins included the CHA (6.9%), right hepatic artery (3.4%), and middle hepatic artery (1.3%) [18].

Beyond these major sources, rare origins of the RGA have also been described. These include direct emergence from the CeT, from a coeliacomesenteric trunk, or from a gastrohepatic trunk [18].

Duplication of the RGA has occasionally been reported, with two arteries arising from the PHA, supplying the stomach independently [18].

These findings underscore the wide anatomical variability of this artery, despite its relatively small caliber [18]. In summary, although the RGA is most often encountered as a branch of the PHA, its origin demonstrates marked variability [18].

### 2.3. Left and Right Gastroepiploic Arteries

The LGEA and RGEA run within the greater omentum and course along the greater curvature of the stomach, where they provide branches to both the gastric wall and the omentum, forming the gastroepiploic arcade [1] (Figure 1).

The RGEA is typically described as the larger of the two vessels. In most cases, it represents the terminal branch of the GDA and proceeds from right to left, accompanied by its adjacent vein, before joining the arcade of the greater curvature. Morphometric studies in cadaveric series have shown an average length of approximately 24 cm and a proximal diameter between 2.6 and 3.0 mm, making it a substantial vessel of the stomach and omentum [19,20]. Variations in its origin are relatively rare. In angiographic and anatomical investigations, the RGEA has been reported to arise directly from the superior mesenteric artery in about 1.5–2% of cases, or less commonly through a common trunk with the middle colic artery in roughly 1% of cases [20]. Exceptional cases have also been described in which the artery originated from the dorsal pancreatic artery—an extremely unusual variant [21], and the absence of the vessel detected preoperatively [22]. Furthermore, the RGEA can provide the infrapyloric artery in 27.6% of the population [23].

The termination of the RGEA is less constant than its origin. While traditional descriptions note a direct anastomosis with the LGEA, radiological and cadaveric studies have demonstrated that this occurs in only a subset of individuals [20,24]. Direct end-to-end anastomosis has been observed in roughly a quarter to a third of cases, whereas in many individuals the anastomosis is represented only by slender or plexiform branches, and in a significant minority there is no true continuity at all [20,24]. Further variability is seen in its omental branches. In a large cadaveric study, Settembre and colleagues identified a particularly prominent branch of the RGEA, which they termed the arteria omentalis magna, present in nearly three-quarters of cases and supplying the midline of the greater omentum [19].

The LGEA, by contrast, arises most consistently from the SA near its terminal portion. It courses from left to right along the greater curvature, contributing gastric branches to the fundus and omental branches to the greater omentum [1]. Compared to its right-sided counterpart, it is generally of smaller caliber, and in some radiographic series it has even been reported as absent in a small proportion of cases [20]. Rare but important variations have been documented [25]. These include unusual duplication, in which a single trunk from the SA divides into two LGEAs that both course toward the stomach, one anastomosing with the RGEA and the other breaking up into finer gastric and omental branches. In some instances, the LGEA may also follow an intrapancreatic course before emerging to supply the stomach or may arise from polar branches of the splenic artery [25].

The pattern of anastomosis between the right and left gastroepiploic arteries is one of the most variable features of their anatomy, yet surgical and clinical significant. Tomioka et al. [24], in a gross cadaveric study, observed direct anastomosis in 94% of specimens, with a robust arterial arch present in about 70% and more delicate mesh-like connections in roughly a quarter. Only a small minority, less than 6%, exhibited complete independence without anastomosis [24]. These findings contrast with earlier radiological studies that suggested much higher rates of incomplete or absent anastomosis, likely reflecting differences in methodology and sensitivity of detection [24].

Taken together, the available evidence indicates that the gastroepiploic arteries, though usually predictable in their origins from the GDA and SA, display a range of anatomic variations.

### 2.4. Other Constant and Inconstant Arterial Branches

Beyond the major vessels supplying the stomach, several smaller arteries provide additional perfusion to the gastric walls. Some of these, such as the short gastric arteries, are relatively constant, while others, like the posterior gastric artery, are more inconstant and variable in origin and presence.

The short gastric arteries usually number between four and seven, arising from the splenic artery or its terminal branches [1]. They pass through the gastrosplenic ligament to supply the fundus of the stomach. They are generally present in all individuals, although their number and size vary considerably [26].

A more variable vessel is the posterior gastric artery (PGA), which provides blood supply to the posterior wall of the upper body and fundus. The prevalence of this artery has been a subject of debate, with early reports ranging from as low as 4% to as high as 99%. A meta-analysis of 38 studies comprising over 3300 subjects found a pooled prevalence of 57.4% [27]. The PGA most commonly originates as a single vessel from the SA (86.5%) but may also arise from the superior polar branch of the SA (11.8%) or, more rarely, from the LGA, the CeT, or even the left inferior phrenic artery [27]. Loukas et al. [28], in a cadaveric series, found the artery in 81.6% of cases, with origins distributed among the LGA (41.8%), SA (25.5%), both LGA and splenic as double PGAs (22.4%), and directly from the CeT (10.2%) [28]. Tortuosity is common, and in a subset of cases the PGA also provides a small branch to the superior pole of the spleen [28].

Other inconstant arteries have also been described in imaging and surgical series. Aberrant branches of the LGA may course posterior to the esophagus, while accessory gastric arteries may arise from the hepatic or splenic circulation [16,17,29]. Modern CT angiography has revealed that such variations occur in nearly one-third of patients with gastric cancer [16,17]. These include duplications or absences of typical gastric vessels, unusual origins from the superior mesenteric artery, and additional branches from polar splenic arteries [26].

## 3. Venous System of the Stomach and Its Variations

### 3.1. Gastric Veins

**Figure 3:**
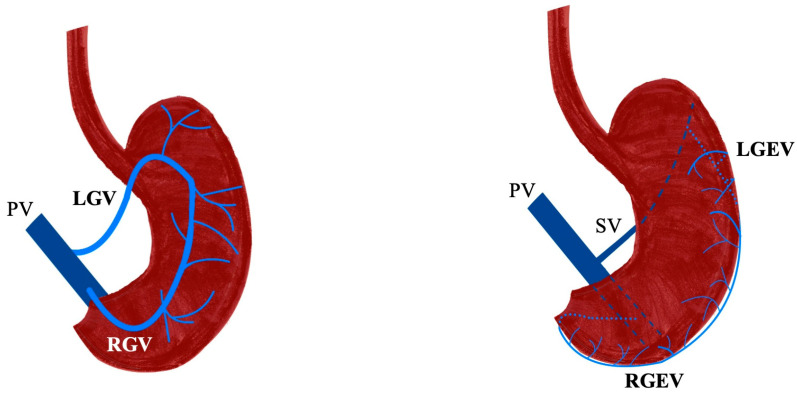
The typical venous drainage of the stomach with the left and right gastric and gastroepiploic veins (LGV, RGV, LGEV, RGEV). PV—portal vein, SV—splenic vein.

The gastric venous system closely parallels the arterial supply of the stomach and drains ultimately into the PV. The two principal vessels are the LGV, also known as the coronary vein, and the RGV, which anastomose along the lesser curvature before draining into the portal venous circulation [1] (Figure 3).

The LGV typically accompanies the LGA, collecting tributaries from the cardia, the lesser curvature, and the distal esophagus. Classically, it drains into the PV, but significant variation has been documented. Large-scale CT and surgical studies classify its course according to its relationship with the major arteries of the CeT. In Lee et al. [30] classification, the LGV may cross anterior or posterior to the CHA (type I), anterior to the LGA (type II), anterior or posterior to the SA (type III), or ascend directly to the proximal PV (type IV) [30]. Most patients have a single LGV, though duplication occurs in 3–4% [30]. Wu et al. [31] proposed a six-type CT-based nomenclature, with the most common being the retro-CHA type (49.8%), followed by pre-splenic (20.6%) and mid-course types (20.0%) [31]. Zhu et al. [32] confirmed that the most prevalent pattern was drainage between the CHA and CeT (58.8%) [32].

LGV variation also involves several aberrant drainage patterns. In some cases, the LGV drains directly into the PV or intrahepatic branches [33]. These aberrant LGV have been classified into three subtypes: type 1, functioning as an accessory portal vein; type 2, with combined intrahepatic and parenchymal distribution; and type 3, with anastomosis into the left intrahepatic portal branches [34]. The prevalence of aberrant LGV is low, estimated at 0.07% in large imaging cohorts [34], while meta-analytic data suggest overall gastric vein variants occur in about 8.3% of the population [33].

The RGV is smaller and drains the pyloric region and the right portion of the lesser curvature. Typically, it empties into the PV, but alternative terminations have been observed. Caty et al. [35] described a rare case of an aberrant RGV that ascended along the common bile duct and drained directly into the porta hepatis, bypassing the PV trunk [35]. Choi et al. [36], in a large CT series of over 2000 patients, reported aberrant RGV in 1.5% of cases, most commonly draining into segment II or III of the liver or directly into intrahepatic portal branches [36].

Taken together, the gastric veins demonstrate notable anatomical variation. While the LGV usually drains into the PV and the RGV into the PV or its confluence, both can exhibit aberrant drainage directly into intrahepatic portal branches, or in the case of the LGV, into the SV.

### 3.2. Gastroepiploic Veins

The venous drainage of the greater curvature of the stomach is provided by the LGEV and RGEV, which course along the greater omentum and ultimately drain into the portal system through different tributaries. Together, they form the gastroepiploic venous arcade, which mirrors the arterial arcade of the same region [1] (Figure 3).

The RGEV is the dominant vessel, draining the right portion of the greater curvature and adjacent omentum. It typically converges with the anterior superior pancreaticoduodenal vein (aSPDV) and empties into the superior mesenteric vein (SMV) near the pancreatic head [37]. However, its termination pattern is highly variable. Cao et al. [37], in a prospective series of 144 patients, identified six types of RGEV confluence: type I, convergence with aSPDV plus a colic vein (36.8%); type II, convergence with aSPDV alone (18.8%); type III, convergence with aSPDV plus both a superior right colic vein and right colic vein (14.6%); type IV, joining a co-trunk with aSPDV and a colic vein (10.4%); type V, convergence with a colic vein only (13.2%); and type VI, direct termination into the SMV without joining other tributaries (6.3%) [37]. These patterns are often grouped into a gastropancreatic trunk (types I–III) and a gastrocolic trunk (types IV–VI), with the former more common in most populations [37].

Further variation is seen in the RGEV course and angle of entry into the SMV/portal vein. Liu et al. [38], using 3D CT reconstructions in 200 patients, classified the terminal segment of the RGEV into large-, middle-, and small-angle groups according to the orientation of the vein as it entered the SMV or PV. About 16% belonged to the large-angle group, 56.5% to the middle-angle group, and 27.5% to the small-angle group [38]. This highlights the wide anatomical diversity in the venous course at the pancreaticoduodenal region [13].

The LGEV drains the left portion of the greater curvature and the omentum, usually converging with the inferior polar veins of the spleen or the SV itself. Compared to the RGEV, its course and confluence are more constant [1]. To the authors’ knowledge, there were no cadaveric or imaging studies recording the LGEV anatomical variations.

### 3.3. Other Constant and Inconstant Venous Branches

Besides the main gastric and gastroepiploic veins, several additional venous tributaries participate in the perigastric drainage. Some of these are relatively constant, while others are inconstant or highly variable [1].

The short gastric veins, usually four to seven in number, accompany the short gastric arteries through the gastrosplenic ligament and drain the fundus of the stomach into the SV [39]. They are considered relatively constant structures, though their number and caliber vary between individuals. In some cases, one or more short gastric veins may be absent, or accessory tributaries may be present, altering the venous return from the gastric fundus [39].

A more inconstant tributary is the posterior gastric vein, which parallels the posterior gastric artery and drains the posterior wall of the upper stomach and fundus. Its frequency is variable across series, and it most often terminates in the SV. In a previous radiological study, its presence has been reported in a minority of cases, with marked variability in size and drainage territory [40].

Another venous confluence of note is the gastrocolic trunk (venous trunk of Henle). First described by Henle in 1868, it represents the confluence of the RGEV with colic and pancreatic tributaries before entering the SMV [41]. A recent systematic review and meta-analysis pooling 38 studies and 2686 subjects found an overall prevalence of 86.9% [41]. The most common configuration, present in more than half of cases, is a gastro-pancreato-colic trunk, in which the RGEV joins the aSPDV and superior right colic veins [41]. Less common arrangements include two-tributary trunks (gastrocolic or gastropancreatic) or atypical tributary patterns. The mean diameter of the trunk is approximately 4.2 mm, and its length, when measurable, is around 10 mm [41].

## 4. Surgical Importance of Stomach Vascular Variations

Stomach vascular anatomy is highly variable, and these variants concentrate along the CeT, hepatic arteries, and the venous confluences that surgeons traverse for lymphadenectomy. Therefore, a consistent principle in gastric surgery is comprehensive preoperative vascular mapping. Routine, patient-specific vascular mapping should precede any oncologic or bariatric gastric operation. Contemporary series show that three-dimensional CT angiography (CTA) or multidetector CT reconstructions delineate arterial and venous routes reliably, lower intraoperative blood loss, and can shorten operative time compared with standard imaging alone [26,40,42].

### 4.1. Implications for Gastric Cancer Gastrectomy

**Figure 4:**
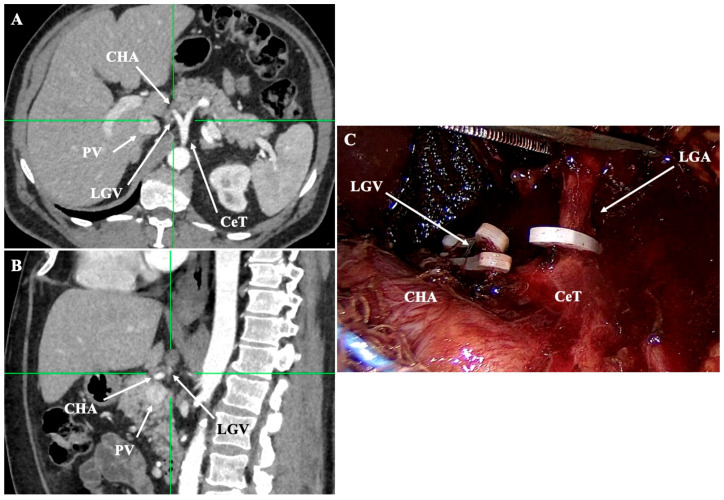
Preoperative CTA (**A**,**B**) and intraoperative view (**C**) demonstrating an LGV running posterior to the common hepatic artery (CHA) before draining into the portal vein (PV). This pattern (type I in Lee et al. [30] classification) is the most common LGV course and is clinically important during station-7 dissection, as early identification reduces the risk of avulsion. Personal archive of Assistant Professor Orestis Lyros.

**Figure 5:**
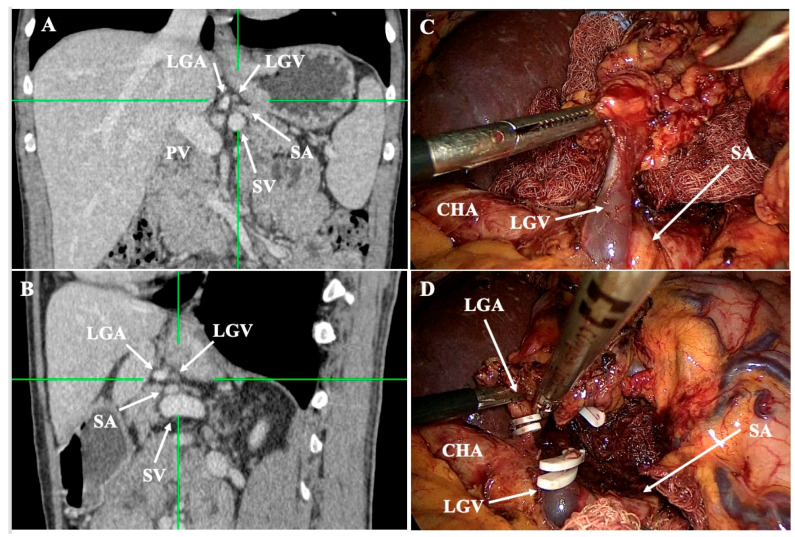
Preoperative computed tomography angiography (CTA) (**A**,**B**) and intraoperative images (**C**,**D**) showing the LGV coursing anterior to the splenic artery (SA). This configuration (type IV) is associated with higher bleeding risk during suprapancreatic dissection and highlights the value of CTA in mapping LGV variants prior to radical gastrectomy [30]. Personal archive of Assistant Professor Orestis Lyros.

**Figure 6:**
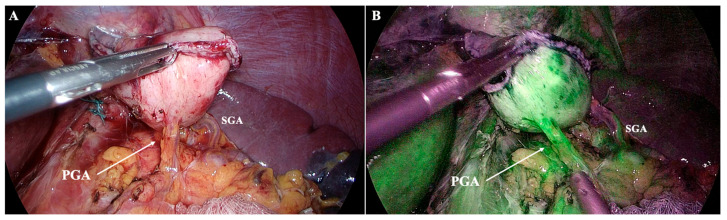
Posterior gastric artery (PGA) preserved during near-total gastrectomy, shown without (**A**) and with (**B**) Indocyanine Green (ICG) fluorescence assessment. ICG confirms robust perfusion of the gastric remnant, demonstrating the functional significance of identifying and preserving the PGA when present. One short gastric artery (SGA) is also visible. Personal archive of Assistant Professor Orestis Lyros.

Radical gastrectomy for stage IB–III disease is built around an oncologically adequate resection with a D2 lymphadenectomy performed in specialized, high-volume centers [43]. Contemporary guidelines define D2 lymphadenectomy as clearance of all D1 stations plus nodes along the CHA, PHA, and SA (excluding the splenic hilum in standard cases), and the CeT; this approach is now the international standard for medically fit patients [43]. Long-term evidence supports this extent of dissection. In the Dutch D1–D2 trial, 15-year follow-up showed lower loco-regional recurrence and fewer gastric-cancer–related deaths after D2 despite higher early morbidity in the era when pancreatico-splenectomy was routine—an excess mitigated by modern spleen- and pancreas-preserving techniques now used in expert centers [44]. Because D2 systematically skeletonizes vessels along the suprapancreatic axis, preoperative vascular road-mapping is pivotal. Modern dual-phase CTA with 3D reconstruction not only delineates the standard vascular routes but also significantly enhances the detection of anatomical variants that influence surgical planning. CTA reliably identifies the course of the LGV relative to the CHA, LGA, and SA [40,42]. This level of preoperative detail reduces intraoperative blood loss, facilitates targeted lymphadenectomy, and prevents inadvertent injury to variant vessels [40,42]. At a population level, MDCT studies in gastric-cancer cohorts consistently demonstrate frequent arterial variations (including non-trifurcation patterns and anomalous hepatic inflow), which increases technical difficulty; targeted planning and energy-device strategy mitigate this risk [16]. Currently, no direct clinical trial has correlated PGA recognition with D2 lymphadenectomy outcomes; however, predominantly arising from the SA system, routine awareness and careful identification of this vessel during fundal dissection are strongly advisable to prevent uncontrolled bleeding and ensure complete nodal clearance [27]. Practical vascular “danger zones” during D2 include:

- Lesser curvature and suprapancreatic corridor (stations 7, 8a, 9, 11p): the LGV most often courses posterior to the CHA or anterior to the LGA; recognizing this pattern helps avoid avulsion during high ligation of the LGA and station-7 dissection [30]. Classify the LGV preoperatively relative to the CHA/SA/pancreas. Intraoperative video-based classification in 217 laparoscopic radical gastrectomy cases found type I (LGV running between the CHA posteriorly and the CA-Figure 4) to be most common (56%), whereas type IV (between SA posteriorly and CA-Figure 5) carried the highest bleeding risk (42%) and was an independent predictor of LGV injury on multivariable analysis [32,45] (Figure 4 and Figure 5). 3D MDCT classifications also emphasize that the LGV may terminate variably into the PV, SV, or their confluence—information that guides safe exposure at the pancreatic head and coeliac axis [40]. When a replaced/accessory left hepatic artery arises from the LGA, D2 dissection must preserve arterial inflow while clearing nodal tissue—an approach illustrated in operative atlases focused on variation-aware D2 technique [6].
- Infrapyloric basin (station 6) and the gastrocolic venous trunk (of Henle): the RGEV exhibits several confluence types with the aSPDV and colic veins; anticipating whether a gastro-pancreatic or gastro-colic trunk is present reduces bleeding during infrapyloric node clearance and kocherisation [37]. A meta-analysis places the venous trunk of Henle (gastrocolic trunk) in ~87% of patients—most commonly as a gastro-pancreato-colic configuration—underscoring why meticulous venous identification matters before dividing the RGEV [41].
- Greater curvature arcade: Variation in the calibre/continuity of the gastroepiploic arterial and venous arcades means the adequacy of greater curvature devascularization should be confirmed visually rather than assumed, especially in subtotal resections aimed at wide margins [16,40].
- Special consideration for PGA presence (approximately 57%): it represents a major inflow to the planned gastric remnant. Lymphatics course along the PGA, but D2 lymphadenectomy does not necessitate PGA division if nodal clearance can be performed while preserving arterial inflow. Preoperative MDCT can easily detect the PGA, while inadvertent ligation has been linked to stump ischemia/leak—use selective preservation and, if divided, confirm stump perfusion intraoperatively [27,28] (Figure 6).

### 4.2. Implications for Bariatric Surgery

Bariatric procedures most relevant to gastric vascular anatomy are laparoscopic sleeve gastrectomy (LSG), Roux-en-Y gastric bypass (RYGB), and biliopancreatic diversion with duodenal switch. LSG and RYGB predominate worldwide, and understanding how constant and inconstant gastric vessels vary—and where they are most likely to be injured or to compromise perfusion—is central to safe technique [46,47].

LSG removes the fundus and most of the greater curvature from approximately 6 cm proximal to the pylorus to the angle of His, leaving a narrow tube based on the lesser curvature [48]. This dissection systematically divides the right and left gastroepiploic arcades and the short gastric arteries, and it approaches the posterior fundal territory where a PGA may be present [48]. The “hot-spot” is the proximal third near the angle of His—an area where vascular variability (short gastrics, LGEA caliber/continuity, and a PGA) can influence tissue perfusion [48].

On the greater-curvature side, several inconstant arterial patterns merit anticipation. The RGEA usually springs from the GDA but may rarely arise from the SMA or even the dorsal pancreatic artery; prominent omental branches are common and can bleed briskly if transected low in the omentum. These variants matter during early lateral mobilization and can be avoided by staying “close to the gastric wall” during dissection [26]. Continuity between the RGEA and the LGEA ranges from a robust arterial arch to only delicate plexiform channels or even no true anastomosis, so the distal body perfusion can depend heavily on residual lesser curvature flow as the sleeve is tubed [29]. Recognizing this variability helps explain why staple-line ischemia clusters proximally when the fundus is skeletonized aggressively [29]. A PGA is present far more often than it was considered (meta-analytic prevalence approximately of 57%), arising most commonly from the SA system but also from the LGA or, rarely, the CeT or left inferior phrenic [27]. Encountering and dividing a sizeable PGA during posterior fundal mobilization can further reduce inflow to the angle-of-His segment [26]. Venous variability is equally critical for blood-loss control. The RGEV drains via multiple patterns into the SMV region—often through gastropancreatic or gastrocolic trunks with the aSPDV and/or colic veins—and its terminal angle of entry varies widely. Awareness of these common confluences and angles helps prevent avulsion near the pancreatic head during greater-omentum division [37,41]. Short gastric veins, variably numerous, empty to the SV and are the typical source of “upper pole” bleeding when the fundus is mobilized [29,40,42].

Modern RYGB creates a small, vertically oriented pouch based on the lesser curvature, excluding the fundus [46]. Pouch perfusion relies chiefly on the left gastric artery (including its esophageal/lesser-curvature branches) and the RGA [46]. Because the RGA origin is highly variable—most often from the PHA but also frequently from the left hepatic, GDA, or CHA [18] aggresive lesser curvature dissection can jeopardize the pouch’s arterial inflow or inadvertently injure hepatic branches if a short RGA arises near the hepatic hilum [46]. Preserving the RGA and the fine LGA branches to the cardia favors robust pouch perfusion [46]. The LGV commonly courses around the CHA or SA before draining to the portal system; prehepatic, presplenic, or mid-course trajectories are all frequent. Gentle handling of the lesser sac around the coeliac axis and hepatogastric ligament avoids troublesome venous bleeding during pouch creation and hiatal work, especially in patients with dilated LGV or duplications [17].

The duodenal switch and its modern single-anastomosis variant rely on a standardized transection of the duodenum just distal to the pylorus, where the GDA serves as a key anatomical landmark [49]. This is clinically relevant because the RGEA typically originates from the GDA. Awareness of this relationship helps avoid inadvertent arterial injury or compromised distal gastric perfusion during the duodenal division [49,50]. As in other bariatric procedures, selective identification of these variant arterial and venous pathways supports safe duodenal transection and reduces bleeding and ischemic complications [50].

### 4.3. Implications for Esophagectomies

Most esophagectomies use a gastric conduit whose perfusion hinges on the RGEA/RGEV, with collateral support from the RGA and the gastroepiploic arcade. Because anastomotic leak (approximately 14%) and conduit necrosis (approximately 3%) remain major causes of morbidity, any factor that compromises this inflow or venous return can translate directly into ischemic complications [51,52]. Notably, prior work has implicated injury to the gastroepiploic vessels, an overly narrow conduit, and hiatal constriction among technical precipitating factors for conduit necrosis—underscoring why variation-aware handling of the gastric vasculature matters during conduit harvest and pull-up [52].

Previous angiographic study in 50 subjects found direct end-to-end RGEA–LGEA anastomosis in only 24% of their sample, with many showing only fine reticular connections or no macroscopic continuity [24]. Conversely, a cadaveric series documented a robust arch in 71% and complete absence in 6% [20]. These conflicting but complementary data argue not to assume a uniform, strong arcade—particularly relevant when the gastric tube apex depends on distal greater curvature collaterals [20,24]. A prospective cohort of 516 patients showed the relative RGEA length (RGEA length/conduit length) independently predicts the anastomotic leak; an RGEA/conduit ratio ≥ 64.69% was protective, supporting pedicle-preserving harvest and maintaining small arterial arcades [53].

**Figure 7:**
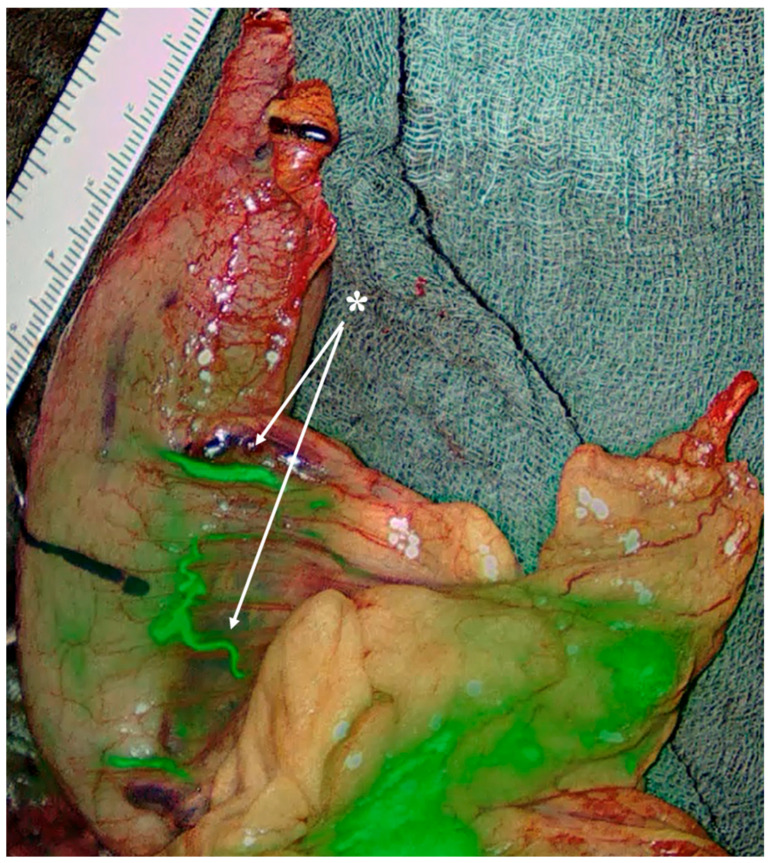
Gastric conduit during Ivor-Lewis esophagectomy evaluated with ICG fluorescence. The preserved right gastroepiploic artery demonstrates adequate perfusion to the conduit tip, illustrating the importance of real-time perfusion imaging, especially in the presence of variable gastroepiploic arcade anatomy *. Personal archive of Assistant Professor Orestis Lyros.

Nevertheless, large studies show anastomotic leak is hard to predict from preoperative factors alone, reinforcing intraoperative perfusion quality as a key, modifiable determinant [52]. Indocyanine green (ICG) fluorescence imaging complements CTA by providing real-time perfusion mapping. ICG allows surgeons to confirm perfusion at critical points such as the gastric conduit tip or the gastric remnant after D2 lymphadenectomy. The visualization the anatomical variants, such as variable gastroepiploic arcade continuity or the presence of a PGA, ICG helps avoid ischemic complications and guides more individualized vascular preservation [52] (Figure 6 and Figure 7). If the RGEA/RGEV are transected, end-to-end microvascular anastomosis with ICG-confirmed patency can allow continuation with a viable gastric conduit, avoiding jejunal/colonic interposition (with its added anastomoses and morbidity) [54].

### 4.4. Implications for Upper Gastrointestinal Bleeding Embolization

Upper gastrointestinal bleeding of gastric origin is usually managed endoscopically; when this fails or rebleeding occurs, targeted trans-arterial embolization is the principal salvage option. Because the stomach’s inflow and outflow are highly variable, effective embolization hinges on patient-specific vascular mapping (dual-phase MDCT/3D reconstructions) to identify the culprit territory and any variant channels that could divert flow or expose non-target organs to ischemia. Imaging series in gastric cohorts show arterial/venous variants in roughly a quarter to a third of patients, supporting routine pre-procedure mapping [11,17,29].

Bleeding along the lesser curvature typically arises from branches of the LGA; however, the LGA origin and branching are frequently variant (non-trifurcation CeT, direct AA origin, or participation in trunks). Pre-embolization review should also screen for hepatic inflow via LGA (accessory/replaced left hepatic artery), as inadvertent embolization risks hepatic ischemia. Variation-aware operative/angiographic frameworks emphasize preserving hepatic inflow when the LGA carries hepatic branches—an equally important caution in trans-arterial embolization planning [11,29].

Hemorrhage from the high fundus/posterior wall often reflects supply from short gastric branches and/or a PGA. The PGA is present far more often than it was considered with a pooled prevalence of approximately 57%, most commonly arising from the splenic artery or its superior polar branch; MDCT has adequate sensitivity to depict it. Failure to recognize a sizable PGA pre-embolization can explain persistent or recurrent bleeding from the posterior fundus after proximal LGA or LGEA occlusion [27].

Bleeding along the greater curvature is usually fed by RGEA and LGEA. The RGEA–LGEA anastomosis ranges from a robust arch to delicate plexiform connections or even no true continuity, so embolization sometimes must be selective (right-sided, left-sided, or both) rather than relying on proximal occlusion alone. Classic and modern series document this variability, which should be anticipated during planning [20,24].

For cardio-fundal and esophagogastric variceal bleeding, the LGV is the dominant inflow. Its course and termination vary—most commonly posterior to the CHA or anterior to the LGA, with occasional direct drainage into intrahepatic portal branches (aberrant LGV). These patterns matter for transvenous/portal interventions (e.g., selective LGV embolization, or portal-directed approaches) and help explain atypical venous filling on angiography [30,31].

Successful embolization in upper gastrointestinal bleeding depends on recognizing the stomach’s common variant channels—notably the LGA (and any hepatic branches), the commonly present PGA to the posterior fundus, the variable gastroepiploic arcade, and the diverse LGV courses for variceal inflow—and tailoring selective catheterization to these individualized maps [27,29,30,31].

## 5. Conclusions

The vascular anatomy of the stomach is highly complex, with frequent arterial and venous variations that hold major implications for surgery and interventional radiology. Consistent landmarks such as the CeT and its branches coexist with inconstant vessels like the PGA and variable gastroepiploic arcades. These patterns influence outcomes in gastrectomy, bariatric surgery, esophagectomy, and embolization for upper gastrointestinal bleeding. High-quality preoperative and preprocedural imaging, especially dual-phase MDCT with 3D reconstruction should be standard to anticipate variants, reduce complications, and optimize patient safety, in line with current international guidance.

## Data Availability

All the data are available upon reasonable request to the corresponding author.

## Abbreviations

The following abbreviations are used in this manuscript:

AA	Abdominal aorta
aSPDV	Anterior superior pancreaticoduodenal vein
CeT	Coeliac trunk
CHA	Common hepatic artery
D2	Second-tier (extended) lymphadenectomy
GDA	Gastroduodenal artery
ICG	Indocyanine green
LGA	Left gastric artery
LGV	Left gastric vein
LGEA	Left gastroepiploic artery
LGEV	Left gastroepiploic vein
LSG	Laparoscopic sleeve gastrectomy
MDCT	Multidetector computed tomography
PHA	Proper hepatic artery
PGA	Posterior gastric artery
PV	Portal vein
RGA	Right gastric artery
RGV	Right gastric vein
RGEA	Right gastroepiploic artery
RGEV	Right gastroepiploic vein
RYGB	Roux-en-Y gastric bypass
SA	Splenic artery
SMV	Superior mesenteric vein
SV	Splenic vein
